# Decline in intertidal biota after the 2011 Great East Japan Earthquake and Tsunami and the Fukushima nuclear disaster: field observations

**DOI:** 10.1038/srep20416

**Published:** 2016-02-04

**Authors:** Toshihiro Horiguchi, Hiroshi Yoshii, Satoshi Mizuno, Hiroaki Shiraishi

**Affiliations:** 1 Center for Environmental Risk Research, National Institute for Environmental Studies, 16-2, Onogawa, Tsukuba, Ibaraki 305-8506, Japan; 2 Research Center for Radiation Emergency Medicine, National Institute of Radiological Sciences, Chiba, Chiba 263-8555, Japan; 3 Nuclear Power Safety Division, Fukushima Prefectural Government, Fukushima, Fukushima 960-8670, Japan

## Abstract

In 2011, 2012, and 2013, in the intertidal zones of eastern Japan, we investigated the ecological effects of the severe accident at the Fukushima Daiichi Nuclear Power Plant that accompanied the 2011 Great East Japan Earthquake and Tsunami. The number of intertidal species decreased significantly with decreasing distance from the power plant, and no rock shell (*Thais clavigera*) specimens were collected near the plant, from Hirono to Futaba Beach (a distance of approximately 30 km) in 2012. The collection of rock shell specimens at many other sites hit by the tsunami suggests that the absence of rock shells around the plant in 2012 might have been caused by the nuclear accident in 2011. Quantitative surveys in 2013 showed that the number of species and population densities in the intertidal zones were much lower at sites near, or within several kilometers south of, the plant than at other sites and lower than in 1995, especially in the case of Arthropoda. There is no clear explanation for these findings, but it is evident that the intertidal biota around the power plant has been affected since the nuclear accident.

Three nuclear reactors of the Fukushima Daiichi Nuclear Power Plant (FDNPP), belonging to the Tokyo Electric Power Company (TEPCO) went into meltdown after the strong earthquake (M 9.0) and subsequent tsunami in eastern Japan in March 2011 (known as the “2011 Great East Japan Earthquake and Tsunami”). Very large amounts of radionuclides were emitted from these reactors to the environment, estimated at hundreds of petabecquerels (PBq)^1^. There are several estimates of the total emissions: TEPCO estimates that 500 PBq of noble gases such as Kr and Xe, 500 PBq of ^131^I, 10 PBq of ^134^Cs, 10 PBq of ^137^Cs and others were emitted into the atmosphere between 12 and 31 March 2011^2^. TEPCO estimated that another 11 PBq of ^131^I as well as 3.5 PBq of ^134^Cs and 3.6 PBq of ^137^Cs were released into the marine environment between 26 March and 30 September 2011, through both atmospheric fallout and direct leakage from the reactors into the sea^3^. The estimates by the Japan Agency for Marine-Earth Science and Technology for the amounts of radionuclides leaked from FDNPP reactors into the sea are greater than those by TEPCO: direct leakage and fallout of ^137^Cs from 21 March to 6 May 2011 were estimated at 4.2–5.6 PBq and 1.2–1.5 PBq, respectively^4^. It is important to recognize that the amount of radionuclides that leaked directly from the reactors into the sea was much more than that entering via fallout. Of course, radionuclides other than ^131^I, ^134^Cs and ^137^Cs, such as ^89^Sr and ^90^Sr, were also emitted from the reactors to the atmosphere and leaked into the marine environment^5^. Even though the 1986 disaster at the Chernobyl Nuclear Power Plant in Ukraine had a much greater release of radionuclides (an estimated total release of 5300 PBq, excluding noble gases)^1^, the Fukushima nuclear reactor accident could have more serious impacts on marine ecosystems because the Chernobyl Nuclear Power Plant was located inland.

There have been several reports on the concentrations of radionuclides such as ^131^I, ^137^Cs and ^90^Sr in the coastal waters of Fukushima^5^,^6^,^7^: concentrations of ^131^I and ^137^Cs in surface seawater near FDNPP in late March to early April 2011 were over 10^5 ^Bq/L and approximately 10^5 ^Bq/L, respectively^7^. On the basis of the ^137^Cs:^90^Sr ratio, the maximum concentration of ^90^Sr might have been 10^4 ^Bq/L^8^. Unfortunately, however, there is less information about the seawater concentrations of radionuclides other than ^131^I, ^137^Cs and ^90^Sr that also leaked from the reactors into the sea. For comparison, the total amount of ^137^Cs emitted to the sea from the Windscale nuclear units (currently, the Sellafield Thermal Oxide Reprocessing Plants) along the west coast of northwestern England from 1952 to 1992 is considered to be 41 PBq, and the maximum annual emission of ^137^Cs was 5.2 PBq in 1975^9^. The Fukushima nuclear disaster released almost the same amount of ^137^Cs as the maximum annual emission from the Sellafield plants (5.2 PBq) into the coastal waters of Fukushima over a relatively short period (from mid-March to early May 2011)^4^, suggesting that the marine organisms around FDNPP, unlike those around Sellafield, might have experienced acute or sub-acute, rather than chronic, exposure to ^137^Cs and other radionuclides.

With regard to possible impacts of radionuclide contamination on ecosystems, it is known that wildlife, including invertebrates, generally has a high tolerance to gamma radiation. For example, at 100–1000 mGy/d some mortality is expected in larvae and hatchlings of flatfish. Flatfish exposed to 10–100 mGy/d show reduced reproductive success, and those exposed to 1–10 mGy/d show potentially reduced reproductive success owing to reduced fertility^10^. An analysis of calculated dose rates in Fukushima’s most affected areas indicated that more severe impacts were likely in the coastal ecosystem adjacent to FDNPP than in forest ecosystems^11^. It is unknown whether any harmful chemicals leaked into the sea along with the various radionuclides, although leakage of boric acid and hydrazine was possible^12^,^13^.

To evaluate possible adverse effects on marine organisms close to FDNPP and in the surrounding area from harmful substances leaked from FDNPP into the sea (not only radionuclides but also other substances in the cooling water), we performed field surveys in the intertidal zones of eastern Japan. A preliminary field survey was conducted at 16 sites within a 20-km radius of FDNPP, as the area impacted by high radionuclide concentrations and the tsunami, on 14 December 2011 (9 months after the disaster; see Supplementary Table S1). Gastropods (herbivorous and carnivorous snails) and crustaceans (crabs, hermit crabs and wharf roaches) were absent at almost all sites, with the exception of a limited number of small barnacles, mussels and limpets. Among the 16 sites surveyed, only one individual rock shell, *Thais clavigera*, was collected, at Namikura in the town of Naraha (identified as P4 in Supplementary Table S1). These observations were very unusual because these gastropods and crustaceans, as well as the rock shell, are usually observed in intertidal zones all over Japan^14^,^15^.

In April, July and August 2012, we performed detailed field surveys at 43 coastal sites in eastern Japan, not only in Fukushima (as the area impacted by higher radionuclide concentrations) but also in Chiba, Ibaraki, Miyagi and Iwate Prefectures (as areas exposed to lower or much lower radionuclide concentrations), which were also hit by the tsunami^16^ on 11 March 2011 (Supplementary Table S2). Additionally, in May and June 2013, we conducted quantitative surveys of sessile organisms at seven sites (Supplementary Table S2) representative of those used in the 2012 survey in terms of their substrate (i.e., tetrapods or similar concrete structures along the coast for wave protection) as well as their distance from FDNPP. We used a quadrat method to confirm declines in population densities and species numbers in the intertidal zones in the context of possible ecological effects caused by the Fukushima nuclear disaster accompanying the 2011 Great East Japan Earthquake and Tsunami.

## Results

### Surveys in 2012

**Figure 1:**
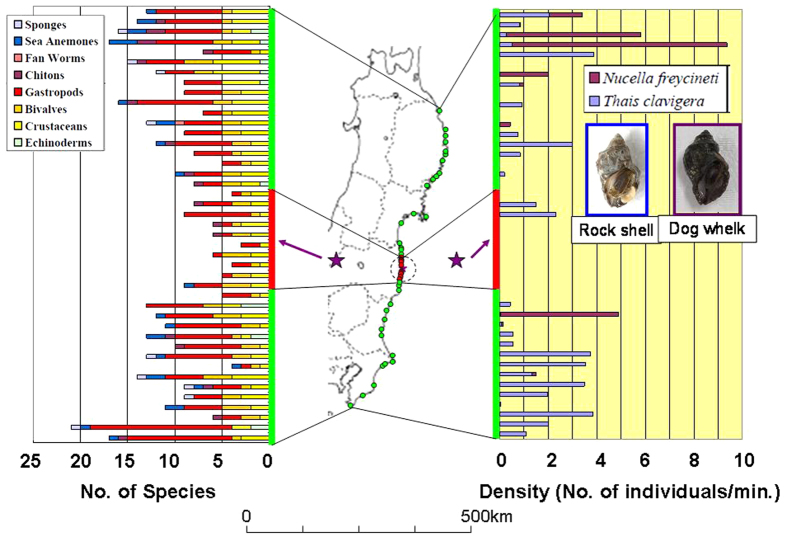
Numbers of intertidal species and the relative population densities of the rock shell (*Thais clavigera*) and dog whelk (*Nucella freycineti*) observed during surveys in 2012 along the coast of northeastern Japan. Dotted circle indicates a radius of 20 km from the Fukushima Daiichi Nuclear Power Plant (FDNPP) in Fukushima Prefecture. Purple star marks the location of FDNPP. Red vertical bars on graphs and red dots on map indicate sites located within the 20-km radius of FDNPP. *The partial map of Japan in this figure was modified by the authors from the map of Japan at the following website: http://www.freemap.jp/item/japan/japan1.html.

The number of animal species observed in the intertidal zones during the 2012 surveys ranged from 3 (Okuma, Fukushima Prefecture) to 21 (Kamogawa, Chiba Prefecture) (Fig. 1; Supplementary Table S2). The number of intertidal species decreased significantly with decreasing distance from the power plant (regression analysis; *P *= 0.000475 for the sites to the south of FDNPP (*n* = 16), *P* = 0.000036 for those to the north (*n* = 18)). The three animal species at Okuma, approximately 1 km south of FDNPP, were a barnacle (*Semibalanus cariosus*) and two herbivorous snails (limpet, *Cellana grata*, and periwinkle, *Littorina (Littorina) brevicula*). The sizes (i.e., shell length or height) of these barnacles and snails at Okuma were very small (see below) and the densities were very low (i.e., approximately 200 per m^2^). Most of the barnacles and periwinkles were around 5 mm or smaller and most of the limpets were around 10 mm—probably young-of-the-year (YOY).

No rock shell (*Thais clavigera*) specimens were found at 8 of the 10 sites in Fukushima Prefecture within a radius of 20 km of FDNPP, surveyed on 24 and 25 April 2012, although specimens were collected at two sites north of FDNPP, namely Ukedo fishing port and Urajiri (identified as Minami-Soma A) (Fig. 1; Supplementary Table S2). All of these sites were hit by the tsunami on 11 March 2011. Because there were also no rock shell specimens collected at Hirono, surveyed on 10 April 2012, the area without rock shells extended from Hirono to Futaba Beach (identified as Futaba B), a distance of approximately 30 km (Fig. 1; Supplementary Table S2). These areas almost overlap within a 20-km radius of FDNPP but are slightly biased to the south (Fig. 1).

At 27 of the 33 sites other than the 10 within a 20-km radius of FDNPP, specimens of carnivorous snails, either rock shell or dog whelk (*Nucella freycineti*), were collected; no carnivorous snails were collected at the other 6 sites (Fig. 1). The 6 sites without carnivorous snails were also hit by the tsunami. They were not, however, geographically continuous but rather were isolated from each other (Fig. 1); this geographical distribution pattern differed from that of sites closer to FDNPP.

**Figure 2:**
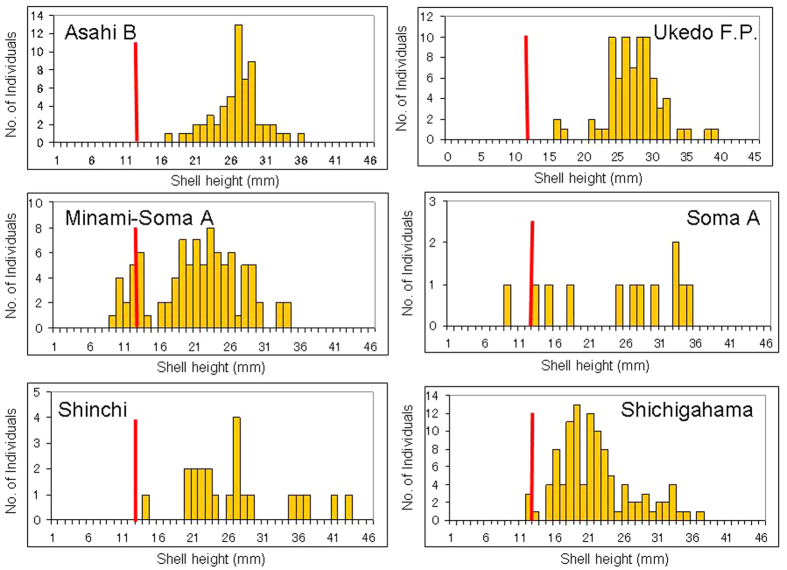
Size distribution of rock shell (*Thais clavigera*) specimens collected in 2012 at representative sites hit by the large tsunami in March 2011. Red bar on each chart represents 12-mm shell height, which is that expected for 1-year-old rock shells^17^.

The shell height distributions of rock shell specimens collected at some sites hit by the March 2011 tsunami (which was more than 5 m high) showed that reproduction and subsequent recruitment of YOY juveniles had occurred in summer and autumn (during the rock shell reproductive season and thereafter) of 2011, even after the earthquake, tsunami and FDNPP accident. This is because some of the specimens had shell heights less than 12 mm and were considered to be YOY (e.g., from Minami-Soma A, Soma A and Shichigahama; Fig. 2) (see Supplementary Fig. S1 for the shell height distributions of rock shell specimens collected at other sites)^16^,^17^.

**Figure 3:**
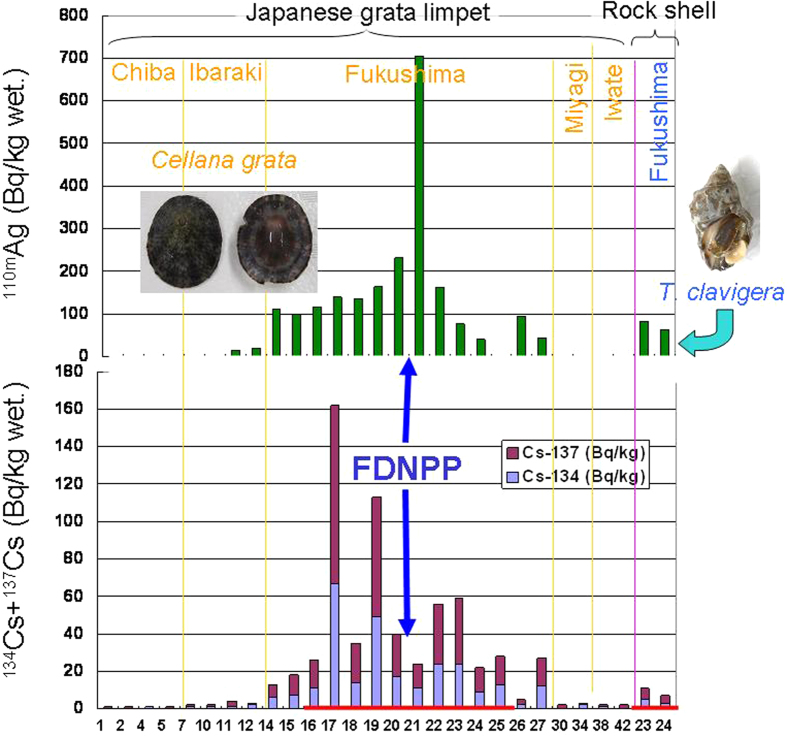
Concentrations of radionuclides in soft tissues of the Japanese grata limpet (*Cellana grata*) and the rock shell (*Thais clavigera*) collected in 2012. Red horizontal lines on x-axis indicate sites located within a 20-km radius of the Fukushima Daiichi Nuclear Power Plant (FDNPP). Numbers on the horizontal axis indicate sampling locations (see Supplementary Table S2).

We determined the concentrations of radionuclides (gamma-emitters) in the soft tissue of the limpet (*C. grata*) and the rock shell (Fig. 3). Concentrations of radio-caesium (sum of ^134^Cs and ^137^Cs concentrations) were higher in the limpet specimens collected at sites in Fukushima Prefecture than in those collected from other prefectures, reflecting generally higher radio-caesium concentrations in seawater in the southern coastal area of Fukushima, possibly due to predominant local water currents^6^,^7^,^9^. Interestingly, radio-silver (^110m^Ag) was also detected in both limpet and rock shell specimens (mainly in those collected at sites in Fukushima Prefecture), and the radio-silver concentrations of these specimens were generally higher than those of radio-caesium (Fig. 3; Supplementary Table S3). The highest concentration of radio-silver was 704 Bq/kg wet-weight in the limpet. The ^110m^Ag:^137^Cs ratios in the limpet and rock shell were 1.5–54.2 and 13.6–15.7, respectively; the respective ^110m^Ag:^134^Cs ratios were 2.1–64.0 and 16.4–20.9 (Supplementary Table S3). The ^110m^Ag:^137^Cs ratios in the limpet and rock shell were generally higher than those in terrestrial and freshwater organisms in Fukushima Prefecture such as spiders, ants, earthworms, frogs, and lizards, as well as those in freshwater crabs and snails^18^. Further study is needed to explain these phenomena, because the transport mechanisms and fate of radio-silver and radio-caesium might differ among terrestrial, freshwater and marine environments. The geographic trend in radio-silver concentrations in the soft tissue of the limpet and rock shell seemed different from that of radio-caesium, possibly implying a different route of contamination. Also, radio-silver concentrations in the soft tissue of the rock shell were significantly different from those of radio-caesium (one-way ANOVA; *P* = 0.023, *n* = 2), but in the limpet, those of radio-silver were not significantly different from those of radio-caesium (one-way ANOVA; *P* = 0.053, *n* = 26). This may reflect differences in ecological characteristics of the rock shell and the limpet, in that rock shells are carnivorous (i.e., they prefer barnacles, oysters and mussels) mainly inhabiting the middle intertidal zone, whereas limpets are herbivorous (preferring microalgae) mainly inhabiting the upper intertidal zone^14^,^17^.

### Surveys in 2013

**Figure 4:**
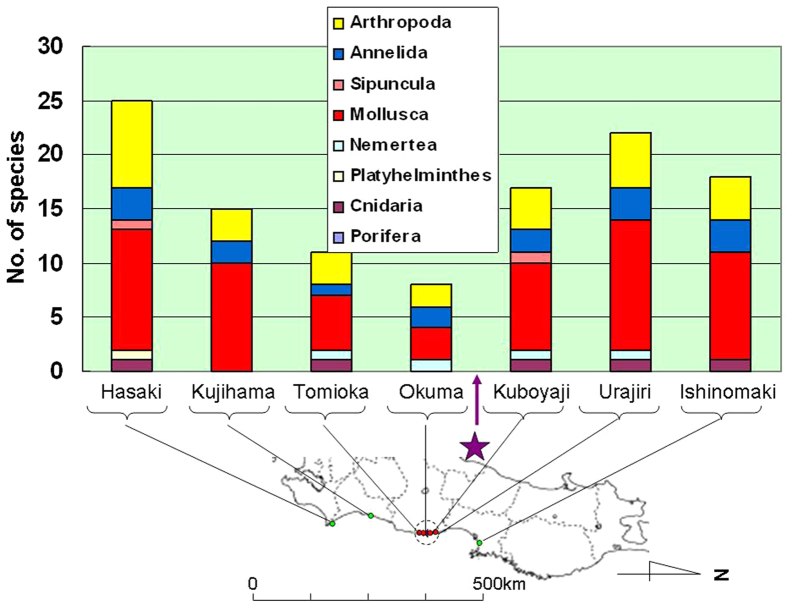
Total numbers of sessile species in intertidal zones, as sampled with a 50-cm × 50-cm quadrat in May–June 2013. Purple star marks the location of Fukushima Daiichi Nuclear Power Plant (FDNPP). Dotted circle on map indicates a radius of 20 km from FDNPP. *The partial map of Japan in this figure was modified by the authors from the map of Japan at the following website: http://www.freemap.jp/item/japan/japan1.html.

**Figure 5:**
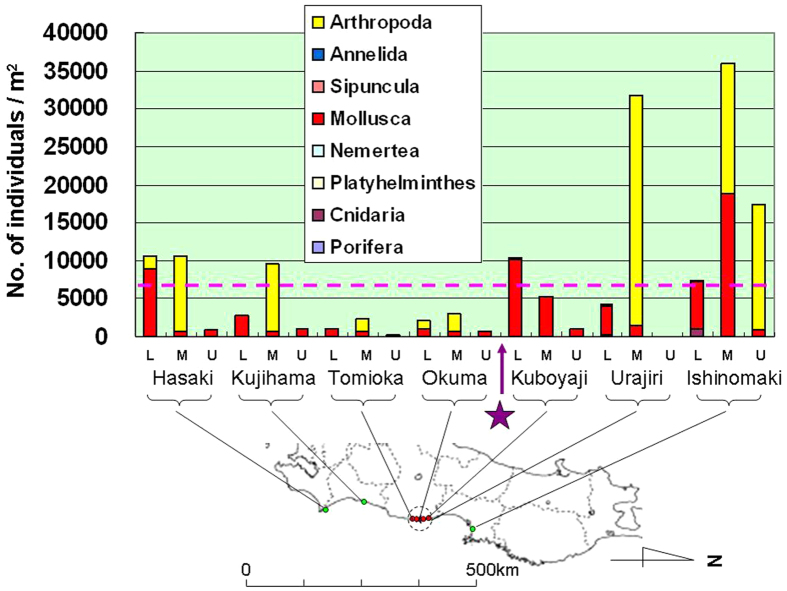
Densities of sessile organisms (number/m^2^) in intertidal zones (by elevation). L: lower intertidal zone; M: middle intertidal zone; U: upper intertidal zone. Data were collected by using a 50-cm × 50-cm quadrat in May–June 2013. Purple star marks the location of the Fukushima Daiichi Nuclear Power Plant (FDNPP). Dotted circle indicates a 20-km radius from FDNPP. Pink dotted line represents the average number of individuals per square meter from surveys of sessile organisms conducted in May 1995 by using a quadrat method at 20 sites along the coast of Fukushima Prefecture^20^. The average population density in 1995 was 7158 individuals/m^2^, consisting of Arthropoda (4593, 64.2%), Annelida (179, 2.5%), Mollusca (2348, 32.8%) and other organisms (38, 0.5%)^20^. *The partial map of Japan in this figure was modified by the authors from the map of Japan at the following website: http://www.freemap.jp/item/japan/japan1.html.

**Figure 6:**
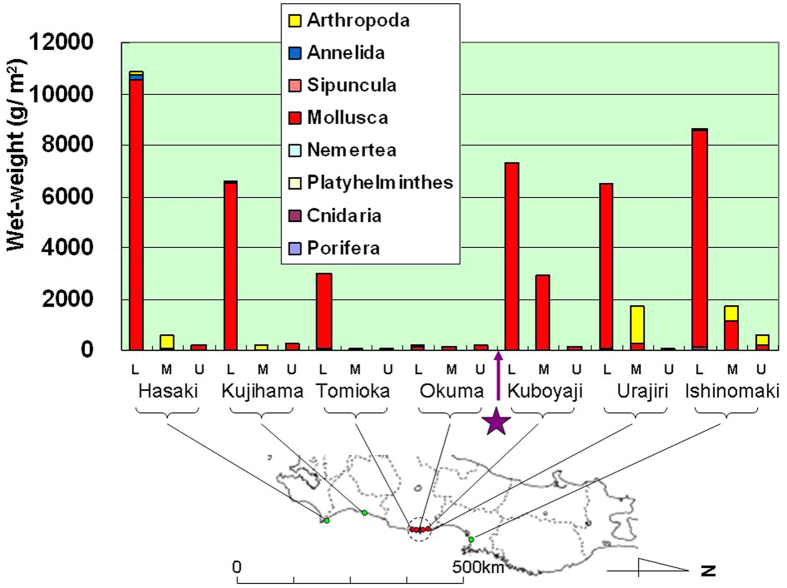
Total wet-weight (g/m^2^) of sessile organisms in intertidal zones (by elevation). L: lower intertidal zone; M: middle intertidal zone; U: upper intertidal zone. Data were collected by using a 50-cm × 50-cm quadrat in May–June 2013. Purple star marks the location of the Fukushima Daiichi Nuclear Power Plant (FDNPP). Dotted circle indicates a 20-km radius from FDNPP. *The partial map of Japan in this figure was modified by the authors from the map of Japan at the following website: http://www.freemap.jp/item/japan/japan1.html.

In 2013 we used a 50-cm × 50-cm quadrat method for quantitative surveys of sessile organisms on the surface of tetrapods or other similar concrete structures placed along the coast for wave protection. Results include the number of species, the number of individuals per square meter, and the wet-weight of whole organisms per square meter (Figs 4, 5, 6, respectively).

In terms of total numbers of sessile species in the intertidal zone, the species composition was dominated by the Mollusca and Arthropoda, followed by Annelida (Fig. 4). The maximum number of intertidal species was 25 at Hasaki Beach (Ibaraki Prefecture), followed by 22 at Urajiri (Minami-Soma A, Fukushima Prefecture). The total number of species seemed to decrease with decreasing distance from FDNPP (regression analysis; *P* = 0.049 for sites south of FDNPP (*n* = 4) and *P* = 0.861 for those to the north (*n* = 3)), and the minimum of 8 species was at Okuma, in Fukushima Prefecture, located approximately 1 km south of FDNPP (Fig. 4). The similarity in species number between Tomioka fishing port, Fukushima Prefecture (identified as Tomioka B) and Okuma, both of which are located south of FDNPP, was high and differed significantly from that between other sites, as determined by analysis of similarity (ANOSIM) of the biotic community structure represented by Bray-Curtis similarity among site groups^19^ (*P* = 0.048, *n* = 7; Supplementary Fig. S2).

Population densities, as indicated by the number of individuals per square meter, were higher in the lower and middle intertidal zones than in the upper intertidal zone. Mollusca (i.e., mussels such as *Mytilus galloprovincialis* and *Septifer virgatus*) and Arthropoda (i.e., barnacles such as *Chthamalus challengeri*) predominated at all sites surveyed (Fig. 5). The maximum population densities (individuals per square meter) at each site were 10,620 (Hasaki Beach, Ibaraki Prefecture), 9468 (Kujihama Beach, Ibaraki Prefecture), 2404 (Tomioka fishing port, Fukushima Prefecture), 2864 (Okuma, Fukushima Prefecture), 10,368 (Kubo-yaji, also identified as Futaba A, Fukushima Prefecture), 31,728 (Urajiri, Fukushima Prefecture) and 35,896 (Ishinomaki, Miyagi Prefecture) (Fig. 5). There was a high level of similarity between the occurrences at Tomioka fishing port and Okuma (ANOSIM; *P* = 0.029, *n* = 7) (Supplementary Fig. S2). Population densities of sessile organisms at Tomioka fishing port and Okuma were less than one-tenth to about one-fourth those at other sites (Hasaki Beach, Kujihama Beach, Urajiri and Ishinomaki). The population density of sessile organisms at Kubo-yaji, located approximately 1 km north of FDNPP, was comparable to those at Hasaki and Kujihama beaches, but approximately one-third those at Urajiri and Ishinomaki. Interestingly, however, the species composition of sessile organisms at Kubo-yaji differed markedly from that at other sites surveyed, in that the Arthropoda accounted for less than 1% of all sessile organisms collected (Fig. 5). Population densities of Arthropoda at sites around FDNPP (Tomioka fishing port, Okuma and Kubo-yaji) were lower than those at other sites (*t*-test; *P* = 0.050, *n* = 7).

Maximum values for total wet-weight, defined as the combined wet-weight of whole organisms per square meter, were found in the lower intertidal zone at each site, followed by the middle and upper intertidal zones (Fig. 6). Mollusca predominated at all sites surveyed, followed by Arthropoda. The maximum total wet-weights (g/m^2^) at each site were 10,851 (Hasaki Beach), 6608 (Kujihama Beach), 2993 (Tomioka fishing port), 169 (Okuma), 7327 (Kubo-yaji), 6478 (Urajiri) and 8628 (Ishinomaki) (Fig. 6). Despite the similarity between Tomioka fishing port and Okuma, both located south of FDNPP (ANOSIM; *P* = 0.029, *n* = 7; Supplementary Fig. S2), the lower intertidal zone of Tomioka fishing port was quite different from all other zones (Fig. 6). The total wet-weights in each intertidal zone at Okuma were much lower than those at other sites. The total wet-weights in the middle and upper intertidal zones at Tomioka fishing port were also quite low and similar to those at Okuma. Although the total wet-weight per square meter in the lower intertidal zone at Tomioka fishing port was higher than that at Okuma, it was still only approximately one-half those at other sites. The total wet-weight in the lower intertidal zone at Kubo-yaji, approximately 1 km north of FDNPP, was similar to those at Kujihama Beach and Urajiri, but less than those at Hasaki Beach and Ishinomaki (Fig. 6).

## Discussion

In 2012, the number of species of intertidal biota declined significantly as the distance between the sampling sites and FDNPP became smaller (*P* = 0.000475 for the sites to the south of FDNPP (*n* = 16), *P* = 0.000036 for those to the north (*n* = 18)). No rock shell (*Thais clavigera*) specimens were collected at eight sites near FDNPP in Fukushima Prefecture in 2012. Because rock shell specimens were collected in 2012 at many sites in Miyagi and Iwate Prefectures, as well as at sites in northern Fukushima Prefecture, where the tsunami also hit (Fig. 2), it is unlikely that the absence of rock shells around FDNPP was caused only by the tsunami. The absence of rock shells at sites close to FDNPP (from Hirono to Futaba Beach, a distance of about 30 km) also suggests that reproduction and recruitment did not occur there, or were less successful, in summer and autumn (the reproductive season and thereafter) in 2011. This is in addition to the possible mortality of almost all individuals living there after March 2011, although it is still unknown why adult rock shells living there disappeared or why rock shells had little or no reproductive success there.

Our quantitative surveys in 2013, conducted at sites with similar concrete structures (i.e., tetrapods) for wave protection, showed that the number of species and the population densities (i.e., the number of individuals and whole wet-weight of organisms per square meter) were significantly lower at sites south of FDNPP than at other sites (regression analysis; *P* = 0.049 (*n* = 4) for the number of species, ANOSIM; *P* = 0.048 (*n* = 7) for the number of species, *P* = 0.029 (*n* = 7) for the population densities). Population densities of Arthropoda were also lower at sites around FDNPP (i.e., Tomioka fishing port, Okuma and Kubo-yaji; *P* = 0.050, *n* = 7 (*t*-test)). These results all suggest that intertidal biota—especially Arthropoda—decreased in abundance around FDNPP after March 2011.

TEPCO conducted similar seasonal surveys by using 30-cm × 30-cm quadrats at 20 sites in intertidal zones along the coast of Fukushima Prefecture in 1995, but only published a summary of their results^20^. In May 1995, there was an average of 7158 individual sessile organisms per square meter, consisting of Arthropoda (4593, 64.2%), Annelida (179, 2.5%), Mollusca (2348, 32.8%) and other organisms (38, 0.5%)^20^. It is therefore clear that Arthropoda predominated but that many other invertebrates were also present in Fukushima Prefecture in 1995, before the nuclear disaster in 2011. From this we conclude that the population densities and numbers of species of sessile organisms in the intertidal zones of Fukushima Prefecture have decreased since March 2011, especially south of FDNPP.

Barnacles (Arthropoda) adhere to rocks or concrete structures (e.g., tetrapods) used for wave protection along the coast. It is therefore difficult to believe that the barnacles disappeared from all of their habitats unless the tsunami scoured all rocks and concrete structures to which they were adhering. Thus, it is also notable that barnacles seem to have disappeared from Kuboyaji, north of FDNPP, since the 2011 nuclear disaster, even though the tetrapods remain. Barnacles accounted for approximately 25% of the number of individual sessile organisms at this site in 1995^20^.

It is evident that there were declines in the numbers of species and the population densities of intertidal biota, including Arthropoda and rock shells, at sites close to FDNPP (especially to the south) after the 2011 Great East Japan Earthquake (regression analysis; *P* = 0.049 (*n* = 4) for the number of species, ANOSIM; *P* = 0.048 (*n* = 7) for the number of species, *P* = 0.029 (*n* = 7) for the population densities). There are several possible causal factors. As already mentioned, it is unlikely that the tsunami was the only causal factor. If the tsunami was not the main cause of the declines near FDNPP, then other causes might include acute or sub-acute toxicities from harmful substances, or other factors associated with FDNPP. For example, the cooling water that leaked from the nuclear reactors directly to the sea between March and April 2011 is estimated to have included various radionuclides and several harmful chemicals^12^,^13^ and therefore could have affected the nearby intertidal biota. More severe effects seem to have occurred along the coast south of FDNPP, and this may be due to the predominant local water currents.

Measured concentrations of ^131^I and ^137^Cs in surface seawater near FDNPP from late March to early April 2011 were reported to be over 10^5 ^Bq/L and approximately 10^5 ^Bq/L, respectively^8^. The ^90^Sr concentration could have reached a maximum of 10^4 ^Bq/L^8^. Rock shells and sessile organisms around FDNPP could have been exposed to these substances during this period. Unfortunately, however, there is less information available about the seawater concentrations of other radionuclides that also leaked from the nuclear reactors into the sea and to which rock shells and sessile organisms around FDNPP could have been exposed. To evaluate the possible impact of radionuclides on marine organisms, therefore, it is first necessary to estimate their concentrations in the environment and then to evaluate the dose rates for intertidal biota around FDNPP immediately after the accident.

Wildlife, including invertebrates, are tolerant of gamma radiation, although some mortality is expected in larvae and hatchlings of flatfish at 100–1000 mGy/d. Reduced reproductive success is observed in flatfish at 10–100 mGy/d, and reduced reproductive success due to reduced fertility is possible at 1–10 mGy/d. Invertebrates, such as crabs, are more tolerant of radiation than are flatfish^10^. Acute lethal doses (LD_50_) are estimated at >100 Gy, 10–25 Gy and 0.16 Gy for marine invertebrates, fish, and fish (salmonid) embryos, respectively. Chronic exposure has yielded no-effect dose rates of 10–30 mGy/h (=240–720 mGy/d) for mortality and 3.2–17 mGy/h (=76.8–408 mGy/d) for reproductive capacity in snails, marine scallops, clams and crabs. The no-effect dose rate for reproduction in fish is 1 mGy/h (=24 mGy/d)^21^.

The United Nations Scientific Committee on the Effects of Atomic Radiation (UNSCEAR) analyzed extensively the relevant data on the effects of radiation on the environment and non-human biota^22^,^23^, concluding that maximum dose rates of less than 400 μGy/h (=9.6 mGy/d) to any individual in aquatic populations of organisms would be unlikely to have any detrimental effects at the population level^24^. This is based on the knowledge that there is little consistent and significant evidence for any effects on reproductive capacity at dose rates <200 μGy/h (=4.8 mGy/d)^25^,^26^,^27^. A generic dose rate of 10 μGy/h (=240 μGy/d) is suggested for use in screening out environmental exposure situations of negligible concern^27^,^28^,^29^.

In contrast, the analysis of calculated dose rates in Fukushima’s most affected areas by Garnier-Laplace *et al*.^11^ suggests that more severe impacts were likely in the coastal ecosystem adjacent to FDNPP than in forest ecosystems. Estimated maximum dose rates for ^131^I, ^134^Cs and ^137^Cs ranged from 210–4600 mGy/d—the lowest for marine birds and the highest for macroalgae (brown algae)—with intermediate values of 2600 mGy/d for benthic biota such as fish, molluscs and crustaceans. Such high dose rates portended marked reproductive effects (and even mortality in the most radiosensitive taxa) in all marine wildlife groups with life history characteristics that confined them to the near-field contaminant release area. This was under the assumption that there were no additional marine releases after March 2011, and in the absence of any estimates of dose rates from other possible radionuclides (e.g., ^58^Co, ^95^Zr, ^99^Mo, ^99m^Tc, ^105^Ru, ^106^Ru, ^129m^Te, ^129^Te, ^132^Te, ^136^Cs, ^132^I, ^140^Ba, ^140^La)^11^.

Another report estimated dose rates (sums of internal and external exposure to ^131^I, ^134^Cs and ^137^Cs) from the compiled arithmetic means of radionuclide concentrations in marine organisms collected at coastal stations in Fukushima from 10 May 2011 to 12 August 2012^30^. The highest estimated dose rates were approximately 0.17–0.25 μGy/h (=4.08–6 μGy/d) for ascidians, macroalgae, sea urchins and holothurians and 0.10–0.17 μGy/h (=2.4–4.08 μGy/d) for benthic fish, crustaceans and molluscs. The dominant contributor to these dose rates was external exposure to ^134^Cs and ^137^Cs; much of any ^131^I present would have substantially decayed. The maximum estimated dose rate for biota was 4.4 μGy/h (=105.6 μGy/d) for benthic fish; this was based on a measurement in fat greenling (*Hexagrammos otakii*) sampled in August 2012^30^.

Similar estimates based on calculated mean internal and external dose rates are reported for a comprehensive dataset consisting of over 500 sediment, 6000 seawater and 5000 biota data points representative of the geographically relevant area during the first year after the Fukushima nuclear accident (May 2011 to August 2012). The standard deviation for the dose rates was typically higher than the average values by a factor of 2–3, masking any discernible time trend^31^.

According to other researchers^32^,^33^,^34^, Garnier-Laplace *et al*.^11^ may have overestimated dose rates by at least one order of magnitude, because the dose rates reported for the first 3 weeks after the accident were based on equilibrium with the maximum water concentrations for all radionuclides reported from water measurements and for all irradiation pathways^6^,^32^. In contrast, Kryshev and Sazykina^33^ and Kryshev *et al*.^34^ might have underestimated their dose rates for marine organisms in the coastal zone near FDNPP, because they did not estimate external dose rates from bottom sediments because the parameters required for such dose rate calculations were not available. It should be noted, moreover, that the dose rates in the above-mentioned reports^30^,^31^,^33^,^34^ were based on benthic species; dose rates for intertidal species such as the rock shell near FDNPP from March to April 2011 might have been higher than these estimates, although there is even less information available to estimate these dose rates.

In order to properly understand the effects of radionuclide exposure on living organisms in the context of an accident, it is essential to distinguish at least two contrasting phases in terms of the level and type of exposure: an acute exposure phase, with a higher dose lasting a few weeks immediately after the accident, and a chronic exposure phase, with a lower dose lasting many years^31^. The acute phase, extending throughout the initial weeks (up to 2–3 months) following the accident, is characterized by the presence of a large quantity of short-lived radionuclides likely to expose living organisms to high dose rates, mainly through external irradiation, with a significant proportion of the dose delivered by beta-emitting radionuclides^31^. The exposure pathway for aquatic organisms is direct exposure to water, which is the receiving medium of the release^31^. During this phase, acute effects are likely to be observed^31^. Acute effects include any notable biological modification occurring within a few days or weeks of absorption of a significant radiological dose, leading to irreversible damage and, eventually, death^31^. The later or chronic phase takes place on a scale of several months to years, during which the contamination levels in the environment change much more slowly^31^. The effects are of many types, but with large uncertainties inherent in the extrapolations required to interpret any ecological significance of the effects observed at different levels of biological organization^31^.

In addition to evaluating dose rates and effects at individual levels, it is also important to assess the impacts of radiation at population, community and ecosystem levels. From this perspective, Bréchignac *et al*.^35^ enumerated the research priorities as follows:

1. “Systems-level research emphasizing interactive responses to radiation exposure, propagation of effects, delayed effects, and resistance and resilience of ecological systems. Each of these could be designed to examine effects at a) population, guild or community levels, or b) systems functions such as primary productivity, decomposition, energy transfer or nutrient flow^35^.”
2. “Additional research at the organism level should be expanded to include representatives of trophic groups not currently included or understudied (e.g., decomposers). There should also be efforts to expand representation of taxa from multiple geographic regions to supplement the current dominance of data from northern temperate systems. Topical research that would be useful would be to develop better understanding of radiation effects that result in adaptation, acclimation, hormesis and epigenetic effects^35^.”
3. “Field studies are needed to calibrate laboratory studies from both the systems and organism levels. In addition to the opportunities at Chernobyl and Fukushima (decidedly different in terms of ecological systems), studies should be undertaken in radionuclide mining areas. In each of these potential study areas, the investigative designs should be based on gradient analyses approaches and not some attempt to compare to ‘reference sites’^35^.”

A Task Group of the International Union of Radioecology has presented the rationale for adding an ecosystem approach to the suite of tools available for managing radiation safety^36^. Bradshaw *et al*.^37^ claim a broad consensus that environmental protection is best served by methods and concepts targeting populations and their interactions with other biota and abiotic components of ecological systems, compared to the International Commission on Radiological Protection Reference Animals and Plants chosen using various taxonomic and practical criteria to serve as points in ecological risk assessments^37^. The radiosensitivity of each reference organism has been documented in literature, in terms of four individual organism-level endpoints (i.e., early mortality, morbidity, reproductive success and mutation frequency). This method, on the basis of traditional toxicology, is to emphasize individual organisms rather than populations or ecosystems^37^. However, the relationships between individual-level responses and population-level impacts of disturbance are tenuous^37^. Interactions among species, as well as life-history differences, physiological requirements and tolerances, could be more important for determining interspecies differences in susceptibility to radiation than differences in radionuclide-specific dose responses^37^. It suggests that ecological knowledge is essential to understanding the responses of populations to radiation^37^.

Together with the various radionuclides, there could have been a few harmful substances in the reactor cooling water that leaked into the sea, such as boric acid and hydrazine. According to one document from the Nuclear Regulation Authority, Japan, approximately 1.7 t of boric acid (as of the end of December 2011) and 110 t of hydrazine (as of the end of March 2012) had been dumped into the reactors and cooling pools for used nuclear fuels^13^. Most of the hydrazine, however, was added to the cooling pools, and because these pools have closed circulation systems it is not considered to have leaked into the sea^13^. The rest of the hydrazine was dumped into the reactors, where it would have been readily decomposed; therefore, it is probably unrealistic to believe that it would have leaked into the sea^13^.

It is useful to briefly review the toxicities of boric acid and hydrazine to aquatic organisms. Boric acid is known to damage the stomach of insects and may also have some toxic effects on their nervous system^38^,^39^,^40^. Besides damaging the stomach, most borate salts are also abrasive to insect exoskeletons^41^. The mechanism of toxicity in animals, however, is still unknown^41^,^42^. The 24-h median lethal concentration (LC_50_; the concentration at which 50% of the test population dies) of borate salts ranges from 4.6 to 150 mg-boron/L for rainbow trout (*Oncorhynchus mykiss*) and bluegill (*Lepomis macrochirus*)^39^. The 48-h LC_50_ for a water flea (*Daphnia magna*) ranges from 133 to 226 mg-boron/L^39^. Marine organisms may be more tolerant than freshwater species to boric acid, because the concentration of boron is much higher in seawater than in freshwater (e.g., 4.5 mg/L in seawater, whereas it is undetectable in river water)^39^,^43^. In contrast, the estimated 48-h LC_50_ of hydrazine to an amphipod crustacean (*Hyalella azteca*) is 40 μg/L and the 96-h LC_50_ in the guppy (*Poecilia reticulata*) is 610 μg/L^44^,^45^. Less information is available about the toxicities of hydrazine to marine invertebrates.

The exposure situation for intertidal organisms in Fukushima could be complex, with many aspects, including various potential direct impacts such as physical harm from the tsunami, and toxicity from chemicals and radionuclides in the massive release immediately after the accident potentially leading to acute effects. Thereafter, there could be continued chronic releases to the sea, barely quantified at present, and ecological effects, for example on interspecific relationships (such as prey–predator relationships or competition for prey organisms and habitat). The effects could also involve intraspecific relationships (competition for prey organisms, habitat and mating partners)^35^,^36^,^37^.

At much higher dose rates, possibly immediately after the accident, differences among taxa in sensitivity to radiation^25^,^46^ could create competitive advantages for resistant organisms within a taxon, and between populations of interacting taxa. This means that life-history traits, responses to a change in resources and generation time all play a role in determining the outcome of radiation effects, in addition to differences in the radiosensitivity of individual organisms. Such higher exposures may exist during or after the accident^37^. It should also be recognized that, particularly at lower doses, ecological factors and variability can be more important than radiation effects. This may make it necessary to adopt a different conceptual methodology to assess ecosystem-level effects. It may require a site-specific assessment of potential disturbances on the ecosystems^37^.

Further studies are therefore needed to clarify the main causal factors for declining intertidal biota around FDNPP, possibly through determining the acute or sub-acute toxicities of various radionuclides, chemicals or other factors, in laboratory experiments. Continued field observations of spatiotemporal changes in the populations of sessile organisms around FDNPP, including rock shell populations, are also necessary to ensure their recovery in the future, and should consider the characteristics of habitats that may influence the distribution of sessile organisms. The focus should be on increasing population densities and reproductive success in terms of active behaviors such as mating and egg-laying and the subsequent successful recruitment of larvae and juveniles. It will also be necessary to conduct both field and laboratory studies to observe and evaluate possible multigenerational effects such as changes in reproductive success resulting from exposure to low-dose radiation and other environmental stressors.

## Methods

### Preliminary survey in 2011

A preliminary survey was conducted at 16 sites within a 20-km radius of FDNPP in Fukushima Prefecture on 14 December 2011, nine months after the disaster, to visually observe the distribution of the rock shell *Thais clavigera* (Supplementary Table S1). The distributions of other intertidal biota, such as molluscs (bivalves, chitons, and herbivorous and carnivorous snails) and crustaceans (barnacles, crabs, hermit crabs and wharf roaches) were also observed.

### Surveys in 2012

In April, July and August 2012, detailed field surveys were conducted at 43 sites along the coast of eastern Japan, not only in Fukushima (as an area receiving higher concentrations of radionuclides) but also in Chiba, Ibaraki, Miyagi and Iwate Prefectures (as areas receiving lower or much lower concentrations of radionuclides), which were also hit by the tsunami on 11 March 2011^16^ (Supplementary Table S2). The surveys were conducted at 10 sites within a 20-km radius of FDNPP in Fukushima Prefecture on 24 and 25 April 2012. Similar surveys were conducted at 33 other sites in Chiba, Ibaraki, Fukushima, Miyagi and Iwate Prefectures in April, July and August 2012. The intertidal species observed were recorded, and all individuals of carnivorous snails, such as the rock shell (*T. clavigera*) and the dog whelk (*N. freycineti*), were collected. The time needed for sample collection (i.e., the number of individuals collected per minute) was also recorded to calculate the relative population density. Shell height of *T. clavigera* was measured with digital calipers to determine its size distribution by site. Concentrations of radionuclides (gamma-emitters) in the soft tissue of the limpet (*C. grata*) and the rock shell (*T. clavigera*) were determined by gamma spectrometry with germanium semiconductor detection (see below). Specimens used for determining radionuclide concentrations were kept in a freezer at −20 °C until measurement.

### Surveys in 2013

Surveys of sessile organisms were conducted at seven sites in Ibaraki, Fukushima and Miyagi Prefectures in May and June 2013 using 50-cm × 50-cm quadrats (Supplementary Table S2). The sites were representative of those used in the 2012 surveys, in terms of substrate (i.e., tetrapods or similar concrete structures set along the coast for wave protection) as well as distance from FDNPP. Four sites—Tomioka fishing port (Tomioka B), Okuma, Kubo-yaji (Futaba A) and Urajiri (Minami-Soma A)—were located within a 20-km radius of FDNPP in Fukushima Prefecture. The other sites (Hasaki Beach in Ibaraki Prefecture; Kujihama Beach in Ibaraki Prefecture; and Ishinomaki in Miyagi Prefecture) were used as reference sites for comparison. Sessile organisms on the surface of tetrapods or similar concrete structures within a 50-cm × 50-cm quadrat were collected at 3 different elevations in the intertidal zone (lower, middle and upper intertidal zones) at each site. Specimens were preserved in 10% formaldehyde neutral buffer solution. After the species had been identified, the number of individuals and wet-weight were determined for each species at each sampling location and elevation at each survey site.

### Quantification of radionuclides

Concentrations of radionuclides (gamma-emitters) such as radio-caesium (^134^Cs and ^137^Cs) in the soft tissues of limpet and rock shell were analyzed by using a gamma spectrometer with a germanium semiconductor detector (GMX45P-76, Seiko EG&G Ortec, Tokyo, Japan). Gamma-ray emissions were measured at energies of 604.7 keV (^134^Cs), 661.6 keV (^137^Cs) and 884.7 keV (^110m^Ag). Detection times were 16,800–60,000 s and 50,000 s for limpet and rock shell samples, respectively. All tissues of limpets and rock shells were kept at −20 °C until measurement. Whole soft tissues (without sex discrimination) were used as composite samples for the determination of gamma-ray species (Supplementary Table S4).

### Data analysis

We performed regression analysis to test if the number of species in the intertidal zones differed significantly with distance from FDNPP in the 2012 and 2013 surveys. Analysis of variance (ANOVA) was used to evaluate the significance of differences in concentrations of ^110m^Ag, ^134^Cs and ^137^Cs in soft tissues of the limpet and rock shell among sites in the 2012 survey. To assess similarity in the biotic community structure of the intertidal zones in the 2013 survey, we conducted cluster analysis (group average method) on Bray-Curtis similarity matrices for the number of species, and for population density in terms of the number of individuals per square meter and whole wet-weight per square meter. Population density data from lower, middle and upper intertidal zones were merged within each site, and log(1 + *x*) transformed for calculation of Bray-Curtis similarity. Site grouping was performed with a cut-off similarity level of 80%. Differences in the biotic community structure represented by Bray-Curtis similarity among site groups^19^ was tested by analysis of similarity (ANOSIM). Possible differences in population densities of Arthropoda (i.e., the number of individual per square meter) at sites around FDNPP (i.e., Tomioka fishing port, Okuma and Kubo-yaji) compared to those at other sites (i.e., Hasaki Beach, Kujihama Beach, Urajiri and Ishinomaki) were evaluated using a *t-*test under the assumption that they had approximately equal variances in the 2013 survey. Statistical analyses were performed with Microsoft Excel statistical software, except for cluster analysis and ANOSIM, which were conducted using the PRIMER6 software^47^.

## Additional Information

**How to cite this article**: Horiguchi, T. *et al*. Decline in intertidal biota after the 2011 Great East Japan Earthquake and Tsunami and the Fukushima nuclear disaster: field observations. *Sci. Rep.* **6**, 20416; doi: 10.1038/srep20416 (2016).

## Supplementary Material

Supplementary Information
