# Supplementary material for: Decline in intertidal biota after the 2011 Great East Japan Earthquake and Tsunami and the Fukushima nuclear disaster: field observations
Source: Sci Rep. 2016 Feb 4;6:20416. doi: 10.1038/srep20416 (PMC4740796; doi:10.1038/srep20416)
Supplement: Supplementary Information [file srep20416-s1.pdf]

Supplementary Information

Decline in intertidal biota after the 2011 Great East Japan Earthquake and Tsunami and the Fukushima nuclear disaster: field observations

Toshihiro Horiguchi[1,*], Hiroshi Yoshii[2], Satoshi Mizuno[3] and Hiroaki Shiraishi[1]

1. Center for Environmental Risk Research, National Institute for Environmental Studies, 16-2, Onogawa, Tsukuba, Ibaraki 305-8506, Japan
2. Research Center for Radiation Emergency Medicine, National Institute of Radiological Sciences, Chiba, Chiba 263-8555, Japan
3. Nuclear Power Safety Division, Fukushima Prefectural Government, Fukushima, Fukushima 960-8670, Japan

Supplementary Table S1. Site locations for preliminary survey on 14 December 2011

No.	Town/City	Name of site	Latitude	Longitude
P1	Naraha	Yamadahama	37°15'18.19"N	141°00'48.13"E
P2	Naraha	Ide	37°16'26.29"N	141°01'02.37"E
P3	Naraha	Shimo-shigeoka	37°16'39.63"N	141°01'05.20"E
P4	Naraha	Namikura	37°18'25.23"N	141°01'28.21"E
P5	Tomioka	Kegaya	37°19'37.91"N	141°01'35.53"E
P6	Tomioka	Tomioka fishing port	37°20'17.37"N	141°01'40.92"E
P7	Okuma	Kumagawa	37°23'14.32"N	141°02'02.81"E
P8	Okuma	Koirino	37°23'26.78"N	141°02'04.98"E
P9	Okuma	Ottozawa	37°24'22.54"N	141°02'00.57"E
P10	Futaba	Kuboyaji	37°26'42.21"N	141°02'11.07"E
P11	Futaba	Futaba Beach	37°27'12.11"N	141°02'16.91"E
P12	Namie	Ukedo fishing port	37°28'51.55"N	141°02'35.58"E
P13	Namie	Tanashio-motomachi	37°29'34.57"N	141°02'20.04"E
P14	Minami-Soma	Urajiri	37°31'55.57"N	141°01'47.89"E
P15	Minami-Soma	Tsunobeuchi	37°32'57.71"N	141°01'43.08"E
P16	Minami-Soma	Murakami	37°33'09.31"N	141°01'43.66"E

Fukushima Daiichi Nuclear Power Plant is located between Ottozawa (P9) and Kuboyaji (P10). All sites were located within a 20-km radius of the power plant.

Supplementary Table S2. Locations for sampling intertidal biota during surveys in 2012 and 2013.

2012 Survey	2013 Survey	Sampling location				
Date	Date	No.	Prefecture	Name of site	Latitude	Longitude
12-Apr	-	1	Chiba	Shirahama	34°54'11.4"N	139°52'25.4"E
12-Apr	-	2	Chiba	Kamogawa	35°07'13.6"N	140° 08'15.9"E
7-Apr	-	3	Chiba	Taito fishing port	35°19'47.1"N	140°24'13.0"E
13-Apr	-	4	Chiba	Asahi A (Nakayari)	35°40'53.2"N	140°38'37.3"E
13-Apr	-	5	Chiba	Asahi B (Iioka)	35°41'32.0"N	140°43'38.9"E
7-Apr	-	6	Chiba	Inubo	35°41'34.6"N	140°51'51.3"E
13-Apr	25-Jun	7	Ibaraki	Hasaki Beach	35°45'14.8"N	140°50'16.3"E
6-Apr	-	8	Ibaraki	Oarai	36°19'16.0"N	140°35'39.2"E
6-Apr	-	9	Ibaraki	Hiraiso	36°22'04.7"N	140°37'24.4"E
9-Apr	24-Jun	10	Ibaraki	Kujihama Beach	36°30'09.8"N	140°37'48.0"E
9-Apr	-	11	Ibaraki	Kokaigahama Beach	36°39'25.4"N	140°42'38.2"E
9-Apr	-	12	Ibaraki	Otsu	36°50'29.5"N	140°47'52.2"E
10-Apr	-	13	Fukushima	Toyoma	36°59'30.9"N	140°58'37.7"E
10-Apr	-	14	Fukushima	Yotsukura fishing port	37° 06'38.3"N	140°59'58.0"E
10-Apr	-	15	Fukushima	Hirono	37°12'06.1"N	141° 00'10.9"E
24-Apr	-	16	Fukushima	Naraha A (Yamadahama)	37°15'29.1"N	141°00'50.0"E
24-Apr	-	17	Fukushima	Naraha B (Namikura)	37°18'25.2"N	141°01'28.1"E
24-Apr	-	18	Fukushima	Tomioka A (Kegaya)	37°19'40.5"N	141°01'35.1"E
24-Apr	28-May	19	Fukushima	Tomioka B (Tomioka fishing port)	37°20'16.3"N	141°01'45.3"E
24-Apr	26-May	20	Fukushima	Okuma	37°24'21.2"N	141°02'00.4"E
25-Apr	27-May	21	Fukushima	Futaba A (Kuboyaji)	37°26'41.4"N	141°02'10.9"E
25-Apr	-	22	Fukushima	Futaba B (Futaba Beach)	37°27'12.1"N	141°02'20.7"E
25-Apr	-	23	Fukushima	Ukedo fishing port	37°28'51.7"N	141°02'35.6"E
25-Apr	29-May	24	Fukushima	Minami-Soma A (Urajiri)	37°31'33.7"N	141°01'53.8"E
25-Apr	-	25	Fukushima	Minami-Soma B (Tsunobeuchi)	37°32'39.3"N	141°01'43.2"E
11-Apr	-	26	Fukushima	Minami-Soma C (Kitaizumi Beach)	37°39'20.1"N	141°01'11.4"E
11-Apr	-	27	Fukushima	Soma A (Chayagamisaki)	37°45'28.5"N	141°00'02.7"E
11-Apr	-	28	Fukushima	Soma B (Matsukawaura fishing port)	37°49'25.6"N	140°58'23.9"E
30-Jul	-	29	Fukushima	Shinchi	37°52'41.4"N	140°56'01.4"E
30-Jul	-	30	Miyagi	Shichigahama	38°16'60.0"N	141°03'49.7"E
31-Jul	22-Jun	31	Miyagi	Ishinomaki	38°24'49.5"N	141°21'27.3"E
31-Jul	-	32	Miyagi	Onagawa	38°27'54.2"N	141°28'33.9"E
1-Aug	-	33	Miyagi	Minami-Sanriku	38°38'36.6"N	141°26'37.3"E
31-Jul & 1-Aug	-	34	Miyagi	Kesen-numa	38°49'46.0"N	141°36'14.6"E
1-Aug	-	35	Iwate	Rikuzen-Takata A (Wakinosawa)	39°00'21.9"N	141°39'14.7"E
1-Aug	-	36	Iwate	Rikuzen-Takata B (Taiyo fishing port)	38°58'14.1"N	141°40'34.7"E
2-Aug	-	37	Iwate	Otsuchi fishing port	39°21'03.0"N	141°56'03.0"E
2-Aug	-	38	Iwate	Yamada A (Kumagasaki)	39°28'50.5"N	141°59'01.9"E
2-Aug	-	39	Iwate	Yamada B (Myojinzaki)	39°28'43.6"N	142°00'08.0"E
3-Aug	-	40	Iwate	Miyako A (Jodogahama)	39°38'35.3"N	141°58'34.1"E
3-Aug	-	41	Iwate	Miyako B (Taro fishing port)	39°43'57.0"N	141°58'41.9"E
3-Aug	-	42	Iwate	Tanohata	39°54'34.8"N	141°56'58.3"E
4-Aug	-	43	Iwate	Kuji	40°10'43.1"N	141°50'01.1"E

Fukushima Daiichi Nuclear Power Plant is located between Okuma (#20) and Kuboyaji (#21). Red text indicates sites within a 20-km radius of the power plant.

Supplementary Table S3. ^{110m}Ag:^{137}Cs and ^{110m}Ag:^{134}Cs ratios in the limpet *Cellana grata* and the rock shell *Thais clavigera* [a]

2012 Survey	Sampling location for *Cellana grata* specimens[b]			Radionuclide concentrations and ratios in *Cellana grata*				
Date	No.	Prefecture	Name of site	^{110m}Ag (Bq/kg-wet)	^{137}Cs (Bq/kg-wet)	^{134}Cs (Bq/kg-wet)	^{110m}Ag:^{137}Cs	^{110m}Ag:^{134}Cs
12-Apr	1	Chiba	Shirahama	n.d.[c]	1	n.d.	n.d.	n.d.
12-Apr	2	Chiba	Kamogawa	n.d.	1	n.d.	n.d.	n.d.
13-Apr	4	Chiba	Asahi A (Nakayari)	n.d.	n.d.	1	n.d.	n.d.
13-Apr	5	Chiba	Asahi B (Iioka)	n.d.	1	n.d.	n.d.	n.d.
13-Apr	7	Ibaraki	Hasaki Beach	n.d.	1	1	n.d.	n.d.
9-Apr	10	Ibaraki	Kujihama Beach	n.d.	1	1	n.d.	n.d.
9-Apr	11	Ibaraki	Kokaigahama Beach	14	3	1	4.7	14.1
9-Apr	12	Ibaraki	Otsu	19	1	2	19.1	9.6
10-Apr	14	Fukushima	Yotsukura fishing port	110	7	6	15.8	18.4
10-Apr	15	Fukushima	Hirono	98	11	7	8.9	14.0
24-Apr	16	Fukushima	Naraha A (Yamadahama)	117	15	11	7.8	10.6
24-Apr	17	Fukushima	Naraha B (Namikura)	139	95	67	1.5	2.1
24-Apr	18	Fukushima	Tomioka A (Kegaya)	136	21	14	6.5	9.7
24-Apr	19	Fukushima	Tomioka B (Tomioka fishing port)	164	64	49	2.6	3.3
24-Apr	20	Fukushima	Okuma	231	23	17	10.0	13.6
25-Apr	21	Fukushima	Futaba A (Kuboyaji)	704	13	11	54.2	64.0
25-Apr	22	Fukushima	Futaba B (Futaba Beach)	163	32	24	5.1	6.8
25-Apr	23	Fukushima	Ukedo fishing port	76	35	24	2.2	3.2
25-Apr	24	Fukushima	Minami-Soma A (Urajiri)	41	13	9	3.1	4.5
25-Apr	25	Fukushima	Minami-Soma B (Tsunobeuchi)	n.d.	15	13	n.d.	n.d.
11-Apr	26	Fukushima	Minami-Soma C (Kitaizumi Beach)	95	3	2	31.5	47.3
11-Apr	27	Fukushima	Soma A (Chayagamisaki)	44	15	12	2.9	3.7
30-Jul	30	Miyagi	Shichigahama	n.d.	2	n.d.	n.d.	n.d.
31-Jul & 1-Aug	34	Miyagi	Kesen-numa	n.d.	1	2	n.d.	n.d.
2-Aug	38	Iwate	Yamada A (Kumagasaki)	n.d.	1	1	n.d.	n.d.
3-Aug	42	Iwate	Tanohata	n.d.	2	n.d.	n.d.	n.d.

2012 Survey	Sampling location for *Thais clavigera* specimens			Radionuclide concentrations and ratios in *Thais clavigera*				
Date	No.	Prefecture	Name of site	^{110m}Ag (Bq/kg-wet)	^{137}Cs (Bq/kg-wet)	^{134}Cs (Bq/kg-wet)	^{110m}Ag:^{137}Cs	^{110m}Ag:^{134}Cs
25-Apr	23	Fukushima	Ukedo fishing port	82	6	5	13.6	16.4
25-Apr	24	Fukushima	Minami-Soma A (Urajiri)	63	4	3	15.7	20.9

[a]A composite sample was prepared from each station for determination of radionuclides (gamma-emitters) by gamma spectrometry with a germanium semiconductor detection.

[b]Fukushima Daiichi Nuclear Power Plant is located between Okuma (#20) and Kuboyaji (#21). Red text indicates sites within a 20-km radius of the power plant. The location numbers (#) are the same as in Supplementary Table S2.

[c]n.d., not detected or not determined

Supplementary Table S4. Biological specimens used for determination of radionuclides[a]

2012 Survey	Sampling location for *Cellana grata* specimens[b]			*Cellana grata* shell length	
Date	No.	Prefecture	Name of site	Min–Max (mm)	*n*
12-Apr	1	Chiba	Shirahama	28.84–42.93	12
12-Apr	2	Chiba	Kamogawa	25.03–37.04	15
13-Apr	4	Chiba	Asahi A (Nakayari)	13.44–31.88	6
13-Apr	5	Chiba	Asahi B (Iioka)	23.25–45.44	13
13-Apr	7	Ibaraki	Hasaki Beach	20.26–28.58	27
9-Apr	10	Ibaraki	Kujihama Beach	14.29–48.99	29
9-Apr	11	Ibaraki	Kokaigahama Beach	18.52–35.44	46
9-Apr	12	Ibaraki	Otsu	16.13–46.19	38
10-Apr	14	Fukushima	Yotsukura fishing port	25.34–57.44	5
10-Apr	15	Fukushima	Hirono	24.53–45.04	26
24-Apr	16	Fukushima	Naraha A (Yamadahama)	42.01–52.91	7
24-Apr	17	Fukushima	Naraha B (Namikura)	24.84–43.19	23
24-Apr	18	Fukushima	Tomioka A (Kegaya)	27.18–38.66	14
24-Apr	19	Fukushima	Tomioka B (Tomioka fishing port)	25.40–48.83	21
24-Apr	20	Fukushima	Okuma	24.39–33.97	31
25-Apr	21	Fukushima	Futaba A (Kuboyaji)	23.37–38.57	33
25-Apr	22	Fukushima	Futaba B (Futaba Beach)	23.68–38.66	38
25-Apr	23	Fukushima	Ukedo fishing port	44.40 - 53.58	3
25-Apr	24	Fukushima	Minami-Soma A (Urajiri)	21.65–39.48	24
25-Apr	25	Fukushima	Minami-Soma B (Tsunobeuchi)	23.52–34.38	4
11-Apr	26	Fukushima	Minami-Soma C (Kitaizumi Beach)	35.22–46.50	8
11-Apr	27	Fukushima	Soma A (Chayagamisaki)	25.24–31.13	20
30-Jul	30	Miyagi	Shichigahama	34.80–44.78	10
31-Jul & 1-Aug	34	Miyagi	Kesen-numa	25.28–39.10	21
2-Aug	38	Iwate	Yamada A (Kumagasaki)	33.63–44.06	20
3-Aug	42	Iwate	Tanohata	26.58–37.86	20

2012 Survey	Samplig location for *Thais clavigera* specimens			*Thais clavigera* shell height	
Date	No.	Prefecture	Name of site	Min–Max (mm)	*n*
25-Apr	23	Fukushima	Ukedo fishing port	16.80–39.24	30
25-Apr	24	Fukushima	Minami-Soma A (Urajiri)	20.26–32.37	20

[a]A composite sample was prepared from each location for determination of radionuclides (gamma-emitters) by gamma spectrometry with germanium semiconductor detection.

[b]Fukushima Daiichi Nuclear Power Plant is located between Okuma (#20) and Kuboyaji (#21). Red text indicates sites within a 20-km radius of the power plant. Location numbers (#) are the same as in Supplementary Table S2.

Supplementary Figure S1

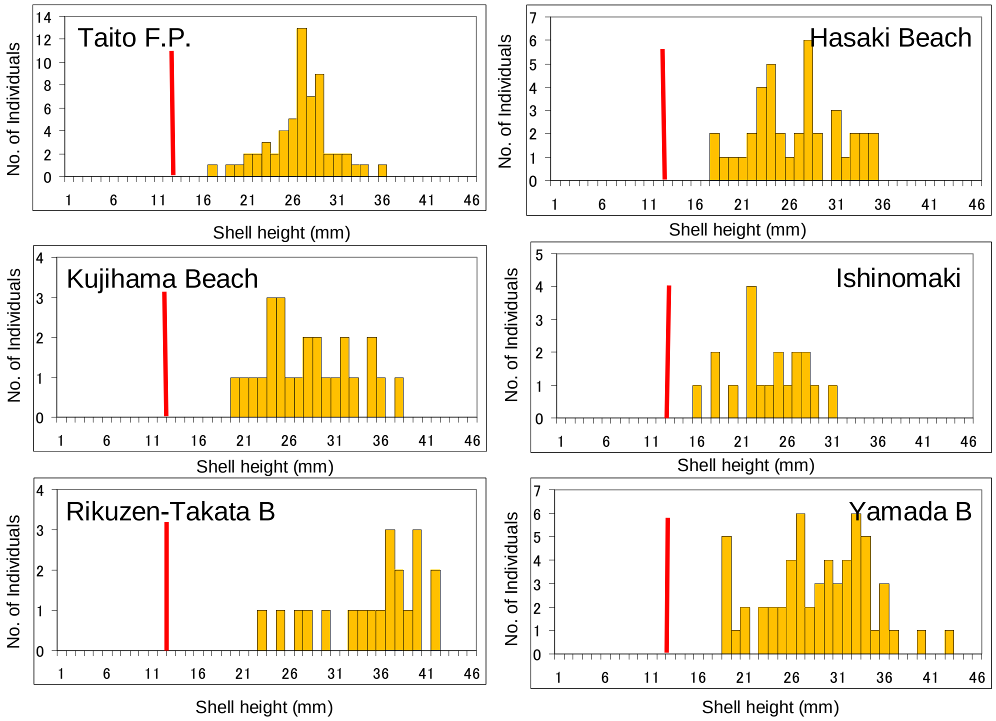

Size distribution of rock shell (*Thais clavigera*) specimens collected in 2012 at sites hit by the tsunami (>3 m) in March 2011. Red bar on each chart represents 12-mm shell height, which is that expected for 1-year-old rock shells [17].

Supplementary Figure S2

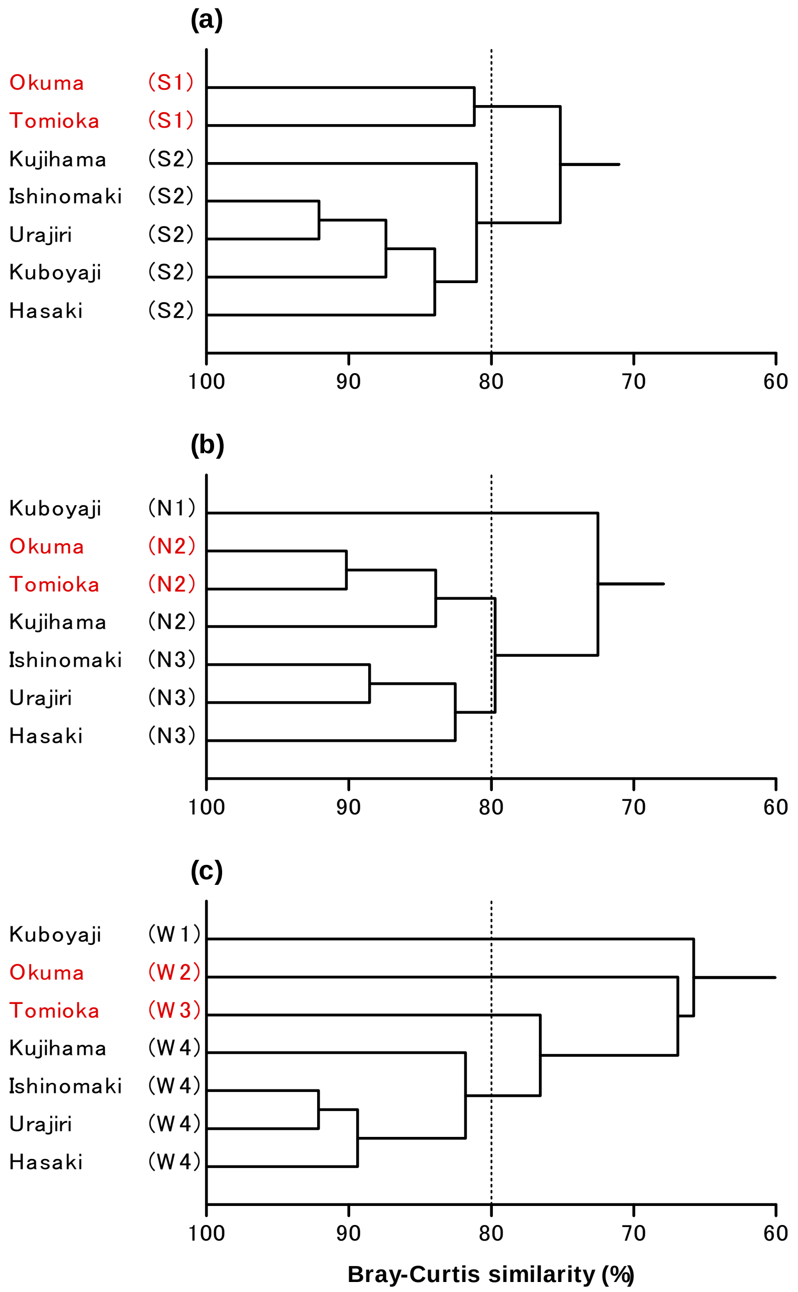

Dendrograms constructed by the group average method on Bray-Curtis similarity matrices for (a) number of species, and population density in terms of (b) number of individuals per m^2 and (c) whole wet-weight per m^2 of intertidal organisms collected in northeastern Japan in 2013. Population density data from lower, middle and upper intertidal zones were merged within each site, and log(1 + *x*) transformed for calculation of Bray-Curtis similarity. Site groupings based on a cut-off similarity level of 80% (dotted lines) are shown as S1-S2, N1-N3 and W1-W4 for panels (a), (b) and (c), respectively. The two sites (Okuma and Tomioka) located south of the Fukushima Daiichi Nuclear Power Plant are shown in red text.
